# A cross-sectional study of Parkinson’s disease and the prodromal phase in community-dwelling older adults in eastern Japan

**DOI:** 10.1016/j.prdoa.2022.100147

**Published:** 2022-05-21

**Authors:** Keita Taguchi, Kazuhiro Iwaoka, Takashi Yamaguchi, Ryota Nozaki, Yuriko Sato, Takahiro Terauchi, Yoshio Suzuki, Kai Takahashi, Kenta Takahashi, Hiroshi Akasaka, Naoki Ishizuka, Tetsuya Maeda

**Affiliations:** Division of Neurology and Gerontology, Department of Internal Medicine, School of Medicine, Iwate Medical University, 1-1-1 Idaidori, Yahaba, Shiwa, Iwate 0283694, Japan

**Keywords:** Parkinson’s disease, Prodrome, Cross-sectional study, Community-dwelling, Older adult

## Abstract

**Introduction:**

Parkinson’s disease (PD) is more prevalent in the aging population, and epidemiological evidence must be constantly updated to provide an accurate understanding of PD prevalence. Various nonmotor symptoms of PD precede the onset of motor symptoms and prodromal PD. The detection of such symptoms is crucial yet remains challenging. In this study, we aimed to clarify the current prevalence of PD and prodromal PD.

**Methods:**

We enrolled 714 community-dwelling older adults (330 men and 384 women) aged ≥ 65 years (mean age 76.3 years). We used a self-administered questionnaire based on the International Parkinson and Movement Disorder Society prodromal PD criteria to obtain information on prodromes and calculate PD probability. Patients with a probability of ≥ 0.3 were considered as having prodromal PD. We analyzed the crude prevalence rates of PD and prodromal PD.

**Results:**

The crude prevalence rate of PD in our sample was 279.7 per 100,000 persons. The crude prevalence rate of prodromal PD and PD probability were 5034.5 per 100,000 persons and 0.057 ± 0.121, respectively. Never smoker (61.4%), physical inactivity (47.0%), regular pesticide exposure (30.7%), and urinary dysfunction (26.5%) were frequent positive prodromes. Subjects with higher PD probability possessed more variable prodromal markers than those with lower probability.

**Conclusion:**

We examined current prevalence rates of PD and prodromal PD in community-dwelling older adults aged ≥ 65 years in Japan. Our questionnaire-based approach to examine prodromal PD provided valuable evidence for the prevalence of prodromal PD in the aging population.

## Highlights

• This study aimed to clarify the recent prevalence rate of PD and prodromal PD. (78/85).
• Questionnaire-based approach was conducted to investigate prodromal PD. (71/85).
• 714 **c**ommunity-dwelling older adults aged 65 or more were enrolled. (66/85).
• Prevalence rate of PD was 279.7 per 100,000 in this study. (58/85).
• Prevalence rate and probability of prodromal PD were 5034.5 and 0.057. (70/85).

## 1 Introduction

Parkinson’s disease (PD) is pathologically characterized by dopaminergic neuronal loss in the substantia nigra (SN) pars compacta and the accumulation of Lewy bodies due to α-synuclein aggregation. When Lewy body pathology extends up the dorsal medulla oblongata toward the SN [1], and dopaminergic neuronal loss in the SN pars compacta exceeds 40% [2], patients exhibit motor symptoms. PD is prevalent in older adults, and the number of PD patients is expected to increase as society ages, which has been described as the PD pandemic [3]. Thus, epidemiological evidence must be constantly updated to gain an accurate understanding of PD prevalence.

PD pathology is already present subclinically before the onset of motor symptoms, and various nonmotor symptoms [4] precede motor symptoms with the progress of pathological findings. Nonmotor symptoms are more likely than motor symptoms to reduce the quality of life of patients [5], [6], [7], [8] and are considered a biomarker of PD. For example, constipation increases the risk of PD by more than four-fold [9], and rapid eye movement sleep behavior disorder (RBD), olfactory loss, and mood disorders have also been shown to heighten the risk of developing PD [10], [11]. In the Parkinson’s At-risk Syndrome study [12], [13], the clinical stages of PD were classified into five stages. The PD and prediagnostic phases are clinical phases in which motor symptoms are present. The subclinical phases are classified into the physiological phase (in which only genetic risk factors are present), the preclinical phase (in which only biomarkers are present), and the premotor phase (in which only nonmotor symptoms are present). The prodromal stage of PD is regarded as the subclinical stage in which motor symptoms defined as parkinsonism are not present but nonmotor symptoms are present [14]. Understanding this stage is crucial for the future development of disease modification therapies.

Prospective cohort studies are considered an effective approach for clarifying the risk factors and etiology of various neurological disorders. The International Parkinson and Movement Disorder Society (MDS) first published criteria for prodromal PD in 2015 [15], which were revised in 2019 [16], comprehensively reviewed the available evidence, and proposed the likelihood ratios of biological markers. The sensitivity and specificity of the criteria for prodromal PD have since been investigated [17], and PD probability can now be calculated using Bayesian methods. The MDS recommended that individuals with a probability of ≥ 0.8 be recruited into clinical studies of disease-modifying therapies and those with a probability of 0.3 to 0.8 be recruited into validation studies of prodromal PD research criteria. We previously retrospectively investigated PD probability in individuals living with PD based on their experience before their diagnosis of PD and revealed that PD probability was 0.123 in the real world [18]. In this study, we used a questionnaire based on the MDS prodromal PD criteria; however, we did not include information on several markers that could be obtained only from individuals who had visited a hospital.

Establishing a method for identifying people at risk of PD before the onset of motor symptoms is crucial yet remains challenging. Although PD patients respond to dopamine replacement therapy, the intervention offers only symptomatic treatment, and fundamental treatments for PD have not yet been developed. Recently, disease-modifying therapy has been proposed as a promising treatment for PD. Several ongoing clinical studies worldwide are examining numerous wet and neuroimaging markers as biological markers of the prodromal stage of PD. However, the application of some of these markers to healthy people who live in the community remains challenging. Although reliable questionnaires and prospective observations are promising procedures in the field of epidemiological research, no epidemiological studies on the prevalence of PD and prodromal PD during the past decade have been conducted in Japan. Therefore, we aimed to clarify the current prevalence rates of PD and prodromal PD in community-dwelling older adults in Japan.

## 2 Methods

### 2.1 Subjects

We enrolled older adults aged ≥ 65 years who were registered in the Yahaba Active Aging and Healthy Brain (YAHABA) study, which was established in 2016 in Yahaba Town, Iwate Prefecture, Japan. The YAHABA study is a community-based prospective cohort study aimed at clarifying the risk factors and etiology of dementia, cerebrovascular disease, and movement disorders in older adults. Physical, cognitive, biochemical, and radiological examinations were performed on all participants, and clinical information, which included current and previous history of illness and medications, biological samples of peripheral blood and urine, and genetic data, was collected, all of which are shared among collaborative prospective studies across Japan on aging and dementia [19]. Baseline registration has been completed, and longitudinal observation is ongoing.

### 2.2 Methods

We registered participants from the YAHABA study who had no cognitive dysfunction or systemic inflammatory or metabolic disorders that hindered their ability to complete the questionnaire. We used a self-administered questionnaire, shown in Table 1, which consisted of 21 items and 18 prodromal markers that were mainly based on the MDS prodromal PD criteria [18]. We obtained participants’ medical records and documents used for regular health checks in the YAHABA study and tabulated the prodromal and biological markers. We defined RBD as the presence of both sleep-related vocalizations and complex motor behaviors of arms and legs during sleep, which is the first criterion of the International Classification of Sleep Disorders criteria, third revision [20]. We made a specific effort to maximize the response rate of participants. Subjects who required assistance in completing the questionnaire or those who required a neurological check-up were advised to visit an outpatient clinic. When a patient was diagnosed with PD, this was recorded in our database. PD probability was calculated in accordance with the MDS prodromal PD criteria, revised edition [16], using the prodromal PD calculator supplied to members as an online program on the MDS website. We defined prodromal PD as a PD probability of ≥ 0.3 in accordance with the MDS declaration of research operating policy.

**Table 1:** Item list of prodrome questionnaire.

No.	Items
1	Regular pesticide exposure
2	Occupational solvent exposure
3	Nonuse of caffein
4	Current smoker
5	Never smoker
6	Former smoker
7	First-degree relative with Parkinson’s disease
8	Type II diabetes mellitus
9	Physical inactivity
10	Low plasma urate levels
11 12	Repeated episodes of sleep-related vocalizations Repeated episodes of sleep-related complex motor behaviors
13	Excessive daytime somnolence
14	Olfactory loss
15	Constipation
16	Urinary dysfunction
17	Erectile dysfunction
18	Neurogenic orthostatic hypotension
19	Symptomatic orthostatic hypotension
20	Depression
21	Global cognitive deficit

### 2.3 Statistical analysis

We calculated the crude prevalence rates of PD and prodromal PD. We divided subjects into two groups: ≥ 0.3 probability of prodromal PD and < 0.3 probability of prodromal PD after excluding PD patients. Clinical background and features of prodromal markers were compared between the two groups using Pearson’s chi-square test. SPSS version 25 (IBM Japan, Tokyo, Japan) was used for all statistical analyses. The significance level was defined as *p* < 0.05.

### 2.4 Ethical aspects

The protocol for this research project was approved by the Ethics Committee of Iwate Medical University (HGH28-12, HG2020-017) and conformed to the provisions of the Declaration of Helsinki. Written informed consent was obtained from all participants.

## 3 Results

### 3.1 Subjects and demographics

The YAHABA study included 962 participants. After excluding patients with dementia and/or serious systemic diseases, 906 participants were enrolled in this study. We received responses from 715 participants, which provided a response recovery rate of 78.9%. One participant had missing age data. Therefore, we enrolled 714 participants, which comprised 330 men and 384 women in the final analysis. The mean age of participants was 76.3 ± 6.23 years (range 68 to 93 years).

### 3.2 Crude prevalence rates of PD and prodromal PD

Of all the subjects, two were diagnosed with PD. The crude prevalence rate of PD was calculated as 279.7 per 100,000 persons. PD probability for the remaining 712 subjects was 0.057 ± 0.121. There were 35 (4.9%) and 677 (95.1%) subjects with ≥ 0.3 and < 0.3 PD probability, respectively, and the probabilities were 0.52 ± 0.16 and 0.033 ± 0.047, respectively. These results indicated that the crude prevalence rate of prodromal PD satisfied our definition and was 5034.5 per 100,000 persons in community-dwelling older adults aged 65 years or older. Among the subjects with ≥ 0.3 probability, two had ≥ 0.8 probability. All values are reported as means ± standard deviations.

### 3.3 Frequent and non-applicable prodromes in community-dwelling older adults

**Fig. 1:**
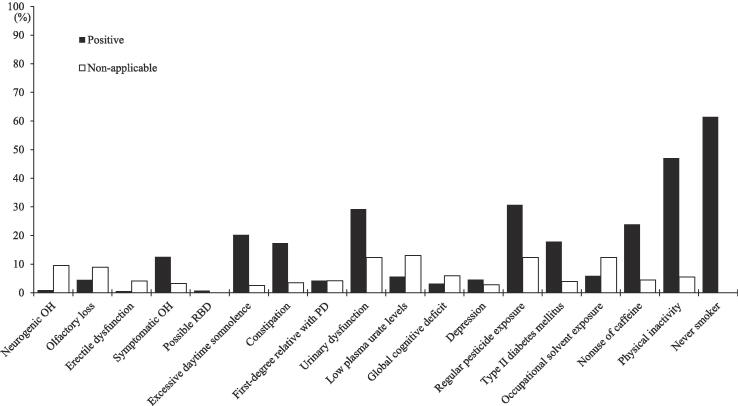
Positive and non-applicable rates of prodromal markers. Positive and non-applicable rates are shown in order of likelihood ratio for the International Parkinson and Movement Disorder Society research criteria for prodromal Parkinson’s disease. MDS, the International Parkinson and Movement Disorder Society; OH, orthostatic hypotension; RBD, rapid eye movement sleep behavior disorder; PD, Parkinson’s disease.

Detailed frequencies of positive and non-applicable prodromes in community-dwelling older adults are shown in Fig. 1. Never smoker (61.4%), physical inactivity (47.0%), regular pesticide exposure (30.7%), and urinary dysfunction (29.2%) were common positive prodromes, whereas low plasma urate level (13.0%), urinary dysfunction (12.3%), regular pesticide exposure (12.3%), and occupational solvent exposure (12.3%) were questionnaire items that were frequently non-applicable or irrelevant to participants.

### 3.4 Differences in demographic and prodrome features between people with ≥ 0.3 and < 0.3 PD probability

There was no significant difference in sex (*p* = 0.379) between subjects with ≥ 0.3 probability and < 0.3 probability; however, there was a significant difference in age (*p* = 0.003).

**Fig. 2:**
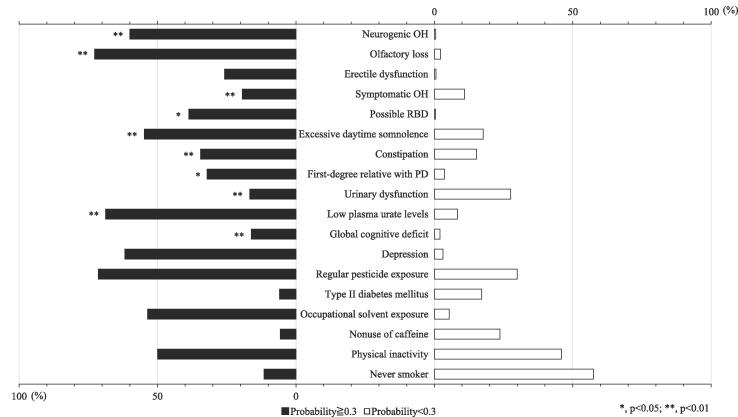
Differences in prodrome features between subjects with higher and lower PD probabilities Community-dwelling older adults with an estimated probability of ≥ 0.3 had significantly higher prevalence rates of several prodromes. OH, orthostatic hypotension; RBD, rapid eye movement sleep behavior disorder; PD, Parkinson’s disease.

Prodromes for current or previous history of medical illness showed significantly higher prevalence in subjects with ≥ 0.3 probability than in those with < 0.3 probability and included head injury (*p* = 0.026), depression (*p* < 0.001), and type 2 diabetes mellitus (*p* < 0.001), as shown in Table 2. Other prodromal markers that showed significantly higher prevalence in subjects with ≥ 0.3 probability than in those with < 0.3 probability were neurogenic orthostatic hypotension (OH; *p* < 0.001), symptomatic OH (*p* < 0.001), olfactory loss (*p* < 0.001), RBD (*p* = 0.010), daytime excessive somnolence (*p* < 0.001), constipation (*p* < 0.001), urinary dysfunction (*p* < 0.001), global cognitive deficit (*p* < 0.001), depression (*p* < 0.001), and having a first-degree relative with PD (*p* = 0.044), as shown in Fig. 2.

**Table 2:** Characteristics of subjects in the community-dwelling older adults.

	All (N = 712)	Probability	
		<0.3(N = 677)	≧0.3(=35)
Sex, male:female, n	329:383	311:366	18:17
Age, mean ± SD, years	76.2 ± 6.53	76.1 ± 6.1	79.3 ± 7.6*
Cerebrovascular disease, n (%)	40 (5.6)	39 (5.8)	1 (2.9)
Malignant neoplasms, n (%)	103 (14.5)	99 (14.6)	4 (11.4)
Head injury, n (%)	57 (8.0)	49 (7.2)	8 (22.9)*
Depression, n (%)	14 (2.0)	9 (1.3)	5 (14.3)**
Diabetes mellitus, n (%)	111 (15.6)	102 (15.1)	9 (25.7)**
D2 blocker medication, n (%)	4 (0.6)	4 (0.6)	0 (0.0)

N, number; SD, standard deviation; PD, Parkinson’s disease; *, p < 0.05 against<0.3 of probability, **, p < 0.01 against<0.3 of probability.

## 4 Discussion

### 4.1 Crude prevalence rates of PD and prodromal PD

We examined the crude prevalence rates of PD and prodromal PD in community-dwelling older adults aged ≥ 65 years. The crude prevalence rate of PD was 279.7 per 100,000 persons. Recently, the prevalence rate of PD was reported at 100–300 per 100,000 persons across all age groups and estimated at 950 per 100,000 persons in older adults aged ≥ 65 years [21]. However, no new studies on the incidence of PD in Japan have been conducted since 2009 [22], and no study has been conducted in eastern Japan. Therefore, this study provides valuable evidence for the prevalence of PD in the aging population in Japan.

Although the crude prevalence rate of prodromal PD is unclear, it is vital for designing clinical trials that specifically investigate disease-modifying therapies. Thus, we aimed to clarify the crude prevalence rate of prodromal PD using the MDS prodromal PD criteria. In this community-based study, the crude prevalence rate was 5034.5 per 100,000 persons when we defined prodromal PD as those with ≥ 0.3 PD probability. The MDS recommends that individuals with a PD probability of ≥ 0.3 be recruited into verification studies of the MDS prodromal PD criteria. Thus, we consider the crude prevalence in our community-based cohort study appropriate. However, the MDS criteria also include other prodromes that we were unable to obtain in our study. These were known gene mutations, SN hyper-echogenicity, polysomnography-proven RBD, clear abnormalities on dopaminergic positron emission tomography (PET)/single-photon emission computed tomography (SPECT), possible subthreshold parkinsonism (unified PD rating scale > 3, excluding action tremor), and abnormal quantitative motor testing. PET has been previously reported as an unreliable biomarker [23], whereas dopamine transporter SPECT imaging, in combination with olfactometry, is currently being used in an ongoing prospective study of PD pathogenesis [13]. However, these large-scale instruments are not applicable to community-based cohort studies. Thus, the development of more broadly applicable detection methods through further research is necessary.

### 4.2 Prodromal PD criteria for cohort studies

In our previous preliminary study [18], we aimed to validate the applicability of the MDS prodromal PD criteria in a community-based cohort by targeting PD patients who had already been diagnosed with PD for several years and retrospectively investigating PD probability during the prodromal stage. In the previous study, we used a questionnaire based on the MDS prodromal PD criteria and enrolled 102 patients with PD. The average probability was 0.123, and 13.4% of patients had a PD probability of ≥ 0.3. In the current study, the PD probability and prodromal PD prevalence rates were lower, at 0.057 and 4.9%, respectively. Moreover, those living with PD had a higher prevalence of olfactory loss, symptomatic OH, constipation, depression, and never smoking, which are considered risk factors of PD and prodromal markers in the MDS research criteria with high likelihood ratios. Together with the results of our previous study, we considered that our questionnaire is acceptable for use in cohort studies.

Of the 712 subjects who completed our questionnaire, two had a PD probability of ≥ 0.8. The MDS recommends patients with a probability of ≥ 0.8 be recruited into disease-modifying or preemptive clinical trials. Given that it is crucial to conduct clinical trials of new potential treatments using appropriate candidates, a simple questionnaire may be valuable for accelerating the development of such treatments. Thus, we aim to continue our prospective cohort study to detect the onset of PD in these two typologies of individuals.

### 4.3 Prodromes latent in older adults

There were numerous prodromes with a high level of evidence in this study, which included never smoker, physical inactivity, regular pesticide exposure, urinary dysfunction, abstaining from caffeine, excessive daytime somnolence, constipation, type II diabetes mellitus, and symptomatic OH. Non-smoking [24], constipation [9], RBD [25], and abstaining from caffeine [26] are well-established risk factors for PD. Furthermore, a meta-analysis reported that the three strongest risk factors for PD are a family history of PD or tremor, constipation, and never smoking, and a history of anxiety or depression, exposure to pesticides, head injury, living in the countryside, taking beta-blockers, working in agriculture, and drinking well water, are relatively weak risk factors for PD [27]. Each of these factors may be associated with the onset of PD, and some may even have multiple environmental risk factors. The Honolulu-Asia Aging study [8] noted that the risk of developing PD is further increased by the existence of multiple nonmotor risk factors as well as a single nonmotor risk factor. Thus, overlapping environmental and nonmotor factors may also increase the risk of developing PD.

### 4.4 Importance of biomarkers

PD is considered a syndrome that encompasses a group of disorders that share a common pathological feature of nigro-striatal dopaminergic denervation. Therefore, judgments based solely on clinical phenotype are inappropriate, and biomarker-proven subtyping of patients with PD is vital for the development of precision medicine in this field [28], [29]. Appropriate biomarkers that can be applied to the wider population to achieve disease modification are expected to emerge in the future.

### 4.5 Limitations

This study has several limitations. First, this was a single-center study. Second, the self-administered questionnaire needs to be improved for use in future studies. For example, the definition of RBD using both sleep-related vocalizations and complex motor behaviors of the arms and legs during sleep should be improved. Third, regular pesticide exposure was particularly common in our cohort because our participants resided in rural areas, which may have influenced our results. Finally, the detection limitations of a questionnaire-based study must be considered, and the results of our study should be interpreted with caution. Our ongoing prospective study will enable the verification of our results in the future.

## 5 Conclusion

We examined the crude prevalence rates of PD and prodromal PD in community-dwelling older adults aged ≥ 65 years in Japan. We used a questionnaire based on the MDS prodromal PD criteria to determine the prevalence of prodromal PD, defined as a PD probability of 0.3, which was easily implemented and deemed useful. These prevalence rates are important for designing clinical trials for novel anti-parkinsonian medication. However, because our questionnaire is a simplified version, its applications may be limited. We recommend further improving our questionnaire for use in community-based cohort studies.

## Funding

This study was supported by Grants-in-Aid from the Japan Agency for Medical Research and Development (JP21dk0207053 and JP21gm1010002). **Role of the funding source** The funder had no role in the design of the study, the collection, analysis, and interpretation of the data, or in the writing of the manuscript.

### CRediT authorship contribution statement

**Keita Taguchi:** Conceptualization, Data curation, Project administration, Formal analysis, Writing – original draft, Writing – review & editing. **Kazuhiro Iwaoka:** Conceptualization, Data curation, Project administration, Formal analysis, Writing – review & editing. **Takashi Yamaguchi:** Data curation, Project administration, Writing – review & editing. **Ryota Nozaki:** Data curation, Project administration, Writing – review & editing. **Yuriko Sato:** Data curation, Project administration, Writing – review & editing. **Takahiro Terauchi:** Data curation, Project administration, Writing – review & editing. **Yoshio Suzuki:** Data curation, Project administration, Writing – review & editing. **Kai Takahashi:** Project administration, Writing – review & editing. **Kenta Takahashi:** Project administration, Writing – review & editing. **Hiroshi Akasaka:** Data curation, Project administration, Writing – review & editing. **Naoki Ishizuka:** Data curation, Project administration, Writing – review & editing. **Tetsuya Maeda:** Conceptualization, Data curation, Project administration, Formal analysis, Writing – original draft, Writing – review & editing.

## Declaration of Competing Interest

The authors declare that they have no known competing financial interests or personal relationships that could have appeared to influence the work reported in this paper.
